# 24 The CD34+ Adipose-derived Stem Cells from Fat Grafts Accelerates Keratinocyte Wound Closure

**DOI:** 10.1093/jbcr/irac012.027

**Published:** 2022-03-23

**Authors:** Stefan Balko, Afshin Raouf, Evan Kerr, Sarvesh Logsetty

**Affiliations:** University of Manitoba, Winnipeg, Manitoba; University of Manitoba, Winnipeg, Manitoba; University of Manitoba, Winnipeg, Manitoba; University of Manitoba, Winnipeg, Manitoba

## Abstract

**Introduction:**

Adipose derived stem cells (ADSCs) possess high regenerative potential and therefore are postulated to enhance re-epithelialization via recruitment of keratinocytes into the wound bed, and thusly improve healing of severe burns. Although ADSC’s are enriched in the stromal vascular fraction (SVF) of fat grafts, the ADSC frequency in these samples is very low (1%) which has hindered their further characterization. So far, the CD34^+^ subset of SVF cells has shown to contain up to 75% of all the ADSCs present in SVF samples but their ADSC frequency remains low (2.5%). Unfortunately, while the ADSC frequency increases in passaged SVF cells, the expression of CD34 is lost in these cells. Therefore, there is a pressing need to identify new ADSC biomarkers to study ADSC mechanisms that accelerate wound closure. In this study, we aim to identify new ADSC biomarkers and to examine the impact of ADSC-enriched cells on skin wound closure.

**Methods:**

Single cells from SVFs were obtained from disassociated fat tissue, collected from excess lipoaspirates used in patients undergoing mastectomies. Native cells (P0), and passaged cells (P3) were placed in cell cultures and the ADSC numbers were obtained using the colony forming unit-fibroblast assays. The expression of 244 cell surface receptors was examined in P0 (low ADSCs) and P3 (high ADSCs) SVF cells to obtain new ADSC biomarkers. In another experiment, the impact of ADSC-enriched CD34^+^ SVF cells on wound closure was assessed. Using transwell inserts containing adult human epidermal keratinocyte (HEKa) cells in the bottom and the bulk SVF or CD34^+^ SVF cells in the top chamber, a scratch was induced in the HEKa cell layer and the scratch area was examined at 0h and 12h.

**Results:**

Our cell surface antibody array experiments revealed 10 potentially new ADSC biomarkers that will be used to examine their ability to enrich for ADSCs. Both the bulk SVF and the CD34^+^ SVF cells showed a 2-fold increase in would closure. It is noteworthy that ADSCs frequency in the CD34^+^ SVF cells is still very low, and these experiments need to be conducted using the new SVF subsets with potentially higher ADSC frequencies based on our cell surface array.

**Conclusions:**

The ADSC-enriched CD34^+^ SVF enhance keratinocyte wound closure, a function that is important to healing of severe burns. Further studies are needed to validate our findings using more purified SVF subsets to identify ADSC-regulated keratinocyte wound closure mechanisms.

